# Supplementary material for: “A friend during troubled times”: Experiences of family caregivers to persons with dementia when receiving professional support via a mobile app
Source: PLoS One. 2022 Aug 2;17(8):e0271972. doi: 10.1371/journal.pone.0271972 (PMC9345357; doi:10.1371/journal.pone.0271972)
Supplement: S1 File — (DOCX) [file pone.0271972.s001.docx]

**INTERVJUGUIDE FÖR INDIVIDUELLA KVALITATIVA INTERVJUER MED ANHÖRIGVÅRDARE TILL PERSONER MED DEMENS**

**Interview guide for individual qualitative interviews with family caregivers to persons with dementia** *(The following questions were used as a guide for the interviews, with probing as necessary)*

**(Efter användning av mobilapplikationen, STAV)**

**(After use of the mobile application, STAV)**

1. Vilken typ av teknisk produkt använder du appen på? Mobiltelefon eller surfplatta? In which type of mobile device do you use the app? Mobile phone or tablet?
2. I vilken miljöer har du använt appen. Hemma i särskilt rum, annan lokal? In which environment have you used the app? At home in a special room, or some other setting?
3. Hur ofta appen används? Per dag eller per vecka? How often have you used the app? Per day or per week?
4. Nu har du provat appen STAV – Stressreducerande app för vårdgivare under 8 veckor, kan du berätta vad du tycker om det? Now that you have used the app STAV over a period of eight weeks, can you describe what you think about it? What was good? What was not so good? Was it easy to use the app? Was it easy to understand?

- Vad har varit bra?
- Vad har varit mindre bra med appen?
- Var det lätt att använda appen? Var den enkel att förstå?

1. Vilka/vilken funktion tycker du var bäst på appen? Varför? Hur ofta har du använt den? Which function of the app did you think was best? Why? How often did you use it?
2. Vad tycker du/ni om innehållet i den applikation som du/ni har prövad? What do you think of the contents of the app? Was the questionnaire easy/difficult/long/complicated? What was your experience of the mindfulness sessions, its length? How did you feel after using mindfulness function? How did you use the diary function? How did you feel about it? Have you used the weblinks? Were there any links that you missed or felt should be removed? Did you register any contacts on the contact list? Was it useful?

- Frågeformulär: (lätt, svår, lång, komplicerad)?
- Mindfulnessfunktioner: Hur upplevde du durationen/längden på sessionerna i den? Hur känner du dig efter mindfulness-sessionerna?
- Dagbok: Hur använde du det? Hur upplevs den?
- Länkar: Har du använt dem? Finns några som du tycker att vi skulle ta bort eller några som fattas där?
- Har du registrerat dina kontakter? Var det värdefullt?

1. Nu skulle jag vilja veta lite mer hur du upplever vår chattfunktion: How did you experience the chat function? Was it easy to understand? Could we help you with advice? Did we write too often or too seldom? Would you have liked to chat with other family caregivers?

Var den lätt att förstå?

Kunde vi hjälpa dig med våra råd?

Skrev vi för ofta, eller för sällan?

Skulle du vilja chatta med andra anhöriga?

1. I vilka situationer har de varit användbara? Har det påverkat ditt vårdande i någon riktning? In which situations has the app been useful? Has it affected your caring of the person with dementia in any way? How?
2. Har ni känt att appen har inverkat på stressnivåer i vårdandet? På vilket sätt i så fall? Have you felt that the app has affected your stress level when caring for your family member with dementia? If so, in what way?
3. Har ni känt att appen har inverkat på er allmänna hälsa? På vilket sätt i så fall? Have you felt that the app has affected your general health? If so, in what way?
4. Har du/ni förslag på förbättring av appens innehåll och användande? Do you have suggestions to improve the contents of the app and its usability?
5. Om appen var offentlig nu, skulle du använda den? Skulle du rekommendera den till andra personer som du känner? If the app is made available for public use, would you use it? Would you recommend others to use it?
6. Finns det något mer som du skulle vilja säga om appen? Would you like to add anything more about the app?
