# Supplementary material for: “A friend during troubled times”: Experiences of family caregivers to persons with dementia when receiving professional support via a mobile app
Source: PLoS One. 2022 Aug 2;17(8):e0271972. doi: 10.1371/journal.pone.0271972 (PMC9345357; doi:10.1371/journal.pone.0271972)
Supplement: Attachment — Submitted filename: Response to Reviewers.docx [file pone.0271972.s002.docx]

| **Questions from the editor** | **Response from authors** |
| --- | --- |
| Include more and longer quotations presenting the words of the interviewee | See response to reviewer 1’s comment below. |
| Provide more information or references about the STAV app | See response to reviewer 1 & 2s’ comments below. |
| Carefully discuss the "gaps of knowledge" behind this work in relation to the relevant literature | The background has been revised to highlight the necessity of providing support to family caregivers of persons with dementia through mHealth solutions. |
| Include a table that displays the sub-themes that emerged, in addition to just listing the six themes | See response to reviewer 1’s comment below. |
| Revise the declaration of intent in the abstract | The abstract has been revised. |
| Better specify the narrative in the introduction | This is done following the reviewers’ advice. |
| Revise the conclusion adding future directions and perhaps the specificities of the Swedish case; - carefully revise the language; -add a few lines explaining the differences between the terms "dementia" and "Alzheimer"; -harmonize the presentation of numbers in the manuscript. | See response to reviewer 1 & 2s’ comments below. |
| Please include additional information regarding the interview guide used in the study and ensure that you have provided sufficient details that others could replicate the analyses. Please include a copy, in both the original language and English, as Supporting Information | The interview guide both in the Swedish and English is added. |
| **Reviewer #1** |  |
| 1. I would like to see more and longer quotations to let the interviewees "speak for themselves" a bit more. I think richer quotations would be helpful in illuminating and in some cases supporting your findings, and in allowing the reader to critically assess your conclusions. | We have added more quotations. However, as mentioned in the paper, interviews from distance are often shorter than face-to-face interviews but contains the same range of topics. |
| 2. Particularly as your study about the basis for your app is unpublished, I would also like to know a lot more about STAV, how it was developed (and any research that it was informed by), what you think is unique about it, and so on. | We have expanded the description of development oft the intervention in the method section. We have also referred to the publication that described how the proposed intervention was to be developed (Kabir et al, 2020). |
| 3. You also state that "not much attention has focused on the physical and mental health of the family members who have to look after their loved ones who have dementia and who take care of them at home." I think this statement needs to be qualified a bit. | The sentence is rewritten. We thank the reviewer for the references on interventions for family caregivers. One of these references has been incorporated in the introduction describing different types of support for family caregivers. |
| 4. I would suggest you include a table that displays the subthemes that emerged, in addition to just listing the six Themes. This would be useful in signposting the discussion for the reader. | A table on data analysis process has been included. As described in the section on data analysis, we did not include subthemes as part of the analytic process. We refer to Braun & Clark’s (2006) article in describing the steps of analysis undertaken in the study. |
| **Reviewer #2:** |  |
| 1. More information or references should be provided about the STAV app. | We have expanded the description of development of the intervention in the method section. We have also referred to the publication that described how the proposed intervention was to be developed (Kabir et al, 2020). |
| 2. The abstract mentions "to evaluate the app's usability". I found this one a rather emphatic declaration, whereas the paper only discusses a few considerations about usablity. I suggest softening the declaration of intent. | It is changed in the manuscript and the abstract by using the word ‘usage’ instead of ‘usability’ which is a more technical word as the reviewer indicates. |
| 3. Some references report studies in specific populations (e.g. ref.2 in Sweden, ref.3 in Europe,...), yet the first paragraph of the introduction is very generic and does not account for geographic differences. I suggest a better level of specification in the narrative. | The introduction now begins with the global picture followed by the European situation on challenges faced by family caregivers in caring for persons with dementia. Thereafter we provide examples of specific challenges for caregivers across countries. |
| 4. At the beginning of the "Intervention" section, the sentence "an overview of available information channels (collection of web links)" needs a verb. | It is corrected. |
| 5. At the beginning of the "Data collection" section, the sentence "it had to be put on hold" should be rephrased as "they had to be put on hold". | It is reformulated. |
| 6. At the beginning of the "Ethical considerations" section, the sentence "Written informed consent were obtained" should be rephrased as "Written informed consent was obtained". | It is corrected. |
| 7. Similar mistakes appear here and there in the text: I suggest a careful language revision. | The manuscript has been thoroughly checked for linguistic errors. |
| 8. I am curious about the chats between nurses and caregivers via the app. Could you provide examples of topics and themes, and the level of detail they were dealt with? | An example of the topic of the chat between the family caregiver and the researcher included how to address behavioral challenges of the person of dementia. However, the chat data was not saved for analysis as we did not have ethical approval for it. |
| 9. I felt the "Conclusion" section was rather meager and generic. I suggest to improve it by also adding future directions, and perhaps the specificities of the Swedish case (if any). | The conclusion has been revised to capture the implications of the specific findings of our study. |
| 10. I am not an expert of neurobiology, therefore I found it difficult to navigate through the terms "dementia" and "Alzheimer", both used in the paper. I guess it could be useful for readers like me to add a few lines explaining the differences. | We have chosen to use the generic term dementia so as not to confuse the readers. |
| 11. Throughout the paper numbers are sometimes written as digits and sometimes as words: please harmonise. | We have followed the following rule: a number is written in words when a sentence begins with a number; in case of single digital number, it is written in words; and in case of larger numbers than single digit they are written as numeric. The manuscript has been checked for mistakes. |
