# Supplementary material for: “A friend during troubled times”: Experiences of family caregivers to persons with dementia when receiving professional support via a mobile app
Source: PLoS One. 2022 Aug 2;17(8):e0271972. doi: 10.1371/journal.pone.0271972 (PMC9345357; doi:10.1371/journal.pone.0271972)
Supplement: Attachment — Submitted filename: Response to Reviewers.docx [file pone.0271972.s003.docx]

| **Reviewer #2:** | **Response from authors** |
| --- | --- |
| The reviewers asked for more information about the STAV app, how it was developed (and any research that it was informed by), what you think is unique about it, and so on. The authors added a generic sentence bearing little or no extra information. | In the description of the intervention on page 4, we have revised to develop the text and inserted the reference of the published paper on how the mobile application was developed. |
| The reviewers asked to improve conclusions by adding future directions and the specificities of the Swedish case. The authors slightly modified one sentence without really tackling the issue. | We have now related the conclusion to the Swedish context. We have reorganized this section to highlight the important features of the app. We have then suggested future directions in research. |
| The reviewers asked to include a table that displays the subthemes that emerged. The authors added a table with a few examples of the data analysis, which I do not find helpful at all. | As described in the section on data analysis, we did not identify subthemes as part of the analytic process. The analytic process followed the recommendation from Braun & Clark, 2008. This included familiarizing ourselves with the text, extracting codes and generating themes. This is also described in the newer publication by the same authors from 2018 |
| I suggest the authors consider the reviewers' comments in a more serious way, if they want to get their paper published in the journal. These are not major revisions and can be done with a little effort. | It had been unintentional if we gave the impression and answered in a way that could be understood as non-serious. We have tried our best to address all comments from all reviewers with complete seriousness. |
